# A Revolution

**Published:** 1875-10

**Authors:** 


### A REVOLUTION.

Our excellent friend, Dr. Lambert, the well-known President of the American Popular Life Insurance Company, whose office is on the corner of Broadway and Canal Street, characterizes a certain important advance which his company has made in the methods of life insurance, as "not merely reform, but Revolution." It is not for the discussion of this point that we now allude to the fact, but only that we may examine the leading features upon which this new system is based.

The first claim made is, that the ability to measure the probable duration of the best human lives, may, by due investigation, be reduced almost to a certainty, and consequently that this class should be set apart from all inferior classes, for insurance at the lowest rates. This is unquestionably sound reasoning and simple justice. The careful, cleanly, healthy, temperate man, naturally classifies himself. He avoids consort with the unclean and the unwholesome. The insurance company which does not encourage this aristocracy of worth, which classifies its risks merely by age, is surely in the wrong. The doubtful man must pay high rates; the worthy and well man pays amply when he pays infinitely less.

Another point is in the kind of policy recommended. This company considers that insurance is usually desired in a time of peril, and that the termination of the peril may well terminate the protection. The usual peril is that the father may leave his young family poor. After a time property accumulates, the family are self-supporting and insurance becomes useless and irksome. So the American Popular recommends a Policy which provides insurance at low cost, just while it is most needed and no longer. Technically, the Policy recommended is the "Term-Life" Policy, and the annual premium demanded comprises the cost of insurance for the year over which the protection is extended. This assimilates life-insurance more nearly to fire and marine insurance than has been the custom heretofore. It does not seek to pile up a reserve with which to meet losses from bad lives or for any future purpose whatsoever. It offers the most insurance for the least cost, and terminates that insurance at the end of the time stipulated.

By this company the greatest possible advantages are given to the healthy man who submits himself to careful and frequent examination to determine his physical state. They declare—what we know and what every physician knows to be true—that the chances of death in any given year after being carefully examined and found in perfect health, are very small. So they rebate the premium, that is, remit a goodly share of the ordinary cost of insurance to those who thus periodically present themselves for examination. Thus, a man of fifty-five, may, if sound, secure an insurance of $10,000 for one year for as small a sum as $60. This is indeed "revolutionary."

Our readers will ask us if we believe this can be done with safety to the guarantors. We answer plainly, we do. We have not preached the doctrines of health and longevity by temperance and right living, so long for nothing. We know that life may be immensely prolonged by these agencies, and that a life-insurance society can afford to protect the class who live purely and temperately, at infinitely small expense, if it will but set this class aside from all the bad-livers, and not ask them to contribute to losses arising from such risks. Readers of this Journal very rarely die, because they know better how to live than the average man and woman, and the few errors they commit are not serious or destructive to health. It would be hard to make us believe that the select and choice people who make up our regular audience, are not better and safer lives for insurance than the average of humans. To this class the company of which we speak offer facilities which they will do well to examine. This they can do by a visit to the office, or by sending a written request for pamphlets. The whole subject is in a nutshell, and will appeal for its inherent consistency to every sensible man.

